# 829. Ease of Aftercare Using a Biosynthetic Wound Matrix for Full-thickness Wound Temporization

**DOI:** 10.1093/jbcr/irag033.268

**Published:** 2026-04-06

**Authors:** Michael R Young, Patrick John Kennedy, Olivia Duru, Nidhi D Aravapalli, Laura K Pezzopane, Beth McGuire, Ariel Rodgers, Nicole P Bernal, John H Loftus

**Affiliations:** The Ohio State Comprehensive Burn Center, Columbus, OH; The Ohio State Comprehensive Burn Center, Columbus, OH; The Ohio State Comprehensive Burn Center, Columbus, OH; The Ohio State Comprehensive Burn Center, Columbus, OH; The Ohio State Comprehensive Burn Center, Columbus, OH; The Ohio State Comprehensive Burn Center, Columbus, OH; The Ohio State Comprehensive Burn Center, Columbus, OH; The Ohio State Comprehensive Burn Center, Columbus, OH; The Ohio State Comprehensive Burn Center, Columbus, OH

## Abstract

**Introduction:**

The standard of care for full-thickness wound temporization has long been using frozen cadaveric tissue. However, in the past year it has been shown that utilizing a temporary biosynthetic wound matrix (BWM) is a safe and effective option for wound temporization. Frozen cadaveric tissue necessitates additional infection-prevention protocols at our institution, whereas the BWM does not. Our aim was to demonstrate the decreased need for advanced wound care and burn treatment room nursing needs when using BWM.

**Methods:**

A retrospective chart review was conducted for the first 9 patients treated in our center. Patients had either acute full-thickness wounds or mixed depth wounds from surgical excision of necrotic burn tissue and were not ready for immediate autografting. After operative debridement, the BWM was secured, and dry sterile dressings with compression wraps were placed. Patient descriptors including total TBSA, treatment TBSA, depth, burn etiology, number of treatment room dressings, LOS, readiness for autografting, and duration of BWM prior to autografting were recorded.

**Results:**

The cohort had an average age of 49 years, with 66% male. Mean TBSA was 11% (range 1-32%), with a mean treated TBSA of 5%. Most patients (77.8%) were inpatients. Burns were 55% mixed depth and 44% full-thickness; etiologies included flame (55%), scald (22%), chemical (11%), and contact (11%). During the first 48-72 hours post-BWM application, no dressing changes were conducted. After BWM adherence, dry outer dressings were replaced if saturated. The average number of dressing changes in the burn treatment room post-BWM application was 2. Upon BWM adherence, 33% were able to shower. The average number of days until graft readiness was 5.7 and until surgery was 6.6 days. The average length of stay was 26.8 days.

Although not directly compared with cadaveric tissue, BWM required minimal advanced dressing changes. Cadaveric tissue aftercare at our institution involves daily dressing changes in the burn treatment room and continuous inpatient care. In contrast, the use of BWM enabled some outpatient management, bedside dressing changes, and showering of patients. With fully booked treatment room schedules, BWM may reduce the aftercare burden.

**Conclusions:**

Our initial experience indicates the ease of aftercare with both burn nursing treatment room requirements and patients’ dressing change frequency post-BWM application.

**Applicability of Research to Practice:**

The BWM should be considered as an alternative to frozen cadaveric tissue for full-thickness and mixed depth wound temporization as it has minimal aftercare requirements without compromising patient care. Future studies should assess the psychological impact of fewer dressing changes with patients and nursing satisfaction.

**Funding for the study:**

N/A.

